# Successful Treatment of *Pantoea agglomerans* Bacteremia Using Oral Antibiotics

**DOI:** 10.1155/2022/6136265

**Published:** 2022-04-23

**Authors:** Megan Penner, Brittany Romans, Lauren Tah, Brianna Argubright, Matthew Strohmeyer

**Affiliations:** ^1^Idaho State University College of Pharmacy at Anchorage, 3211 Providence Dr PSB 111, Anchorage, AK 99508, USA; ^2^Mat Su Regional Medical Center, 2500 S Woodworth Loop, Palmer, AK 99645, USA

## Abstract

A 30-year-old female with a medical history of gastrointestinal reflux and intravenous methamphetamine use was admitted to the hospital with complaints of fever and chills. *Pantoae agglomerans* was isolated in one out of two blood cultures. Although information was limited about the duration and efficacy of oral antibiotics to treat this type of infection, the patient recovered with oral antibiotic treatment following a short course of intravenous antibiotics.

## 1. Introduction

*Pantoea agglomerans* is a Gram-negative facultatively anaerobic bacillus within the *Enterobacteriaceae* family [1]. Formerly named *Enterobacter agglomerans* and *Erwinia herbicola*, this bacterium can be found in fecal material, soil, and plants. Although rare and low virulence, it can be responsible for opportunistic infections in humans via wounds caused by thorns, wooden splinters, or other plant materials that pierce the skin and inoculate blood with the bacteria [2].

*P. agglomerans* is also associated with “cotton fever,” a benign febrile response to endotoxin production following introduction of cotton to the bloodstream [3]. This can occur when cotton is used to filter substances prior to intravenous injection. *P. agglomerans* can affect both immunocompetent and immunocompromised patients, and some immunocompromised patients may also be exposed to the bacteria within the hospital setting where exposure to liquid substances (i.e., intravenous fluid, total parenteral nutrition, propofol, and blood products) or medical equipment contaminated with *P. agglomerans* may occur [4–7]. Recent studies have shown that switching to oral antibiotics after a short course of intravenous (IV) antibiotics for many Gram-negative bacteremia reduces healthcare costs, length of hospital stays, and IV line complications without compromising patient safety outcomes [8, 9]. Although the successful treatment of *P. agglomerans* bacteremia using IV antibiotics has been documented, no reports have been published regarding treatment with oral antibiotics and the defined duration of therapy [10].

## 2. Case Presentation

A 30-year-old Caucasian female with a medical history of anxiety, gastroesophageal reflux disease (GERD), and intravenous methamphetamine and heroin use presented to the emergency department (ED) on four separate occasions. At the first ED visit, the patient presented with epigastric pain and denied UTI symptoms citing completion of a ciprofloxacin course for a urinary tract infection two weeks prior. The urinary pathogen was extended spectrum beta-lactamase *Escherichia coli*, susceptible to fluoroquinolones. The patient acknowledged use of IV methamphetamine that morning. The patient's vitals were normal with the exception of a temperature of 100.4 degrees Fahrenheit. The patient left the ED prior to completion of the evaluation. A urinalysis was obtained which showed eradication of the urinary tract infection. No labs were drawn, and no blood cultures were obtained.

Six days later, the patient returned to the ED with complaints of ongoing abdominal pain and fevers. Two blood cultures were obtained. A comprehensive metabolic panel (CMP) and complete blood count (CBC) were drawn with normal results. The patient was afebrile in the ED, and abdominal symptoms improved after antiemetic administration, so the risks of computerized tomography of the abdomen were noted to outweigh the benefits. The patient was discharged without antibiotics and instructed to follow-up with her primary care provider.

Two days later, the patient was called back to the ED for growth in one of the two blood cultures. The ED physician felt this was likely a contaminant, since the patient remained afebrile in the ED and repeat CMP and CBC were normal. The patient endorsed ongoing nausea, fatigue, and muscle aches and was subsequently discharged with more antiemetics.

Two days later, the blood culture resulted as *P. agglomerans*, and the patient was called back to the ED for possible hospital admission. The patient endorsed ongoing nausea, vomiting, chest pain, body aches, headaches, and fever and was admitted for IV antibiotics and further workup.

### 2.1. Hospital Course

The patient denied recent exposure to plant material or gardening. The patient had a history of GERD but was not currently using acid suppression medications.

The patient was initially treated with IV cefepime 2000 mg q12 h in accordance with the microbiology susceptibility report (Table 1). The patient received one dose in the ED prior to repeat blood cultures being drawn. Abdominal ultrasound was obtained to evaluate the source of the patient's abdominal pain, and results were normal. On day two of the hospital admission, the patient demanded to leave, not citing specific reasons. The patient was educated about and understood the risks of leaving the hospital and discontinuing IV antibiotics, but left against medical advice (AMA). Repeat blood cultures had no growth. Prior to leaving, the patient had completed two full days of IV cefepime. She was prescribed a 12-day course of oral levofloxacin 750 mg by mouth daily to complete a total 14-day antibiotic course.

Approximately two weeks after the patient left the hospital AMA, she was contacted via phone for a follow-up conversation. She stated that she completed the prescribed 12-day course of levofloxacin, and although she still felt general fatigue and malaise, her fever had completely subsided. Idaho State University Institutional Review Board approval and patient consent were obtained for the publication of this case report.

## 3. Discussion

Although the nomenclature has changed multiple times, *P. agglomerans* remains a rare cause of opportunistic infections. The most common conditions caused by this Gram-negative rod include skin and soft tissue infections, osteomyelitis, septic arthritis, and endocarditis, which can all be associated with bloodstream infections [11–15]. Susceptibility tests have been completed in many reports of *P. agglomerans* infections, and the bacteria are often found to be susceptible to amikacin, gentamicin, meropenem, ciprofloxacin, levofloxacin, amoxicillin/clavulanate, and broad-spectrum cephalosporins (i.e., ceftazidime and cefepime). Susceptibility testing is very useful in choosing an antibiotic; however, patients who leave AMA are difficult to treat and clinical judgment must be used to select an oral antibiotic for outpatient treatment.

The lack of evidence for oral treatment of *P. agglomerans* bacteremia has often led to prolonged intravenous treatment [16, 17]. Since the patient refused to complete the inpatient course of IV antibiotics, the pharmacy team researched possible oral options for the patient to continue after leaving the hospital [12, 14, 18]. Based on this research, the decision to initiate oral levofloxacin was made. A twelve-day oral course was also felt to be appropriate in this case, although studies of other uncomplicated Gram-negative bacteremia have shown noninferiority in seven-day courses [19]. Since this complicated patient had an unknown source of bacteremia and was eager to leave the medical center prior to completion of the recommended course of IV antibiotics, a total two-week course was deemed the safest action. Since levofloxacin was noted to have in vitro activity against *P. agglomerans*, the 14-day antibiotic course proved to be adequate as evidenced by absence of further positive blood cultures or fevers [19]. Although many infections with *P. agglomerans* had specific identified sources such as plant materials, blood products, or intravenous fluids, it is unknown how this patient developed such a rare bloodstream infection. No other cultures in the institution grew *P. agglomerans* during this time frame. It was hypothesized that the cause of infection may have been due to intravenous drug use with contaminated needles or cotton filtration. This patient also had GERD, which may have also played a role since GERD and/or antacid use are associated with spontaneous bacteremia [10]. Gastric mucosal injury caused by over acidification of the stomach by GERD or suppression of protective acidity caused by antacid use could lead to the ability of bacteria to move from the stomach into the bloodstream. It is hypothesized that ingestion of unclean fruits and vegetables contaminated with *P. agglomerans* in the presence of mucosal injury might possibly be a cause of bacteremia from this organism [10, 11]. Underlying health conditions such as active malignancy, diabetes mellitus, chronic viral hepatitis, cerebrovascular accident, congestive heart failure, autoimmune or connective tissue diseases, chronic pulmonary obstructive disease, or end-stage renal disease have also been associated with spontaneous bacteremia. However, this patient did not present with any of these other medical conditions [20].

## 4. Conclusion

A two-day course of intravenous cefepime followed by a 12-day course of oral levofloxacin effectively treated *P. agglomerans* bacteremia in a patient with a history of IV drug use and GERD without recurrence. Additional studies are needed to determine if shorter courses of antibiotic therapy would also be efficacious.

## Figures and Tables

**Table 1:** Microorganism susceptibility report.

*Pantoea agglomerans*		
Drug	MIC interpretation	MIC
Ampicillin	I	16
Amp/sulbactam	S	≤4/2
Cefepime	S	≤2
Ceftriaxone	I	2
Levofloxacin	S	≤2
Trimeth/sulfa	S	≤2/38

## Data Availability

No data were used to support this study.
